# Immersive Virtual Reality in Stroke Patients as a New Approach for Reducing Postural Disabilities and Falls Risk: A Case Series

**DOI:** 10.3390/brainsci10050296

**Published:** 2020-05-15

**Authors:** Irene Cortés-Pérez, Francisco Antonio Nieto-Escamez, Esteban Obrero-Gaitán

**Affiliations:** 1 Department of Health Sciences, University of Jaen, Paraje Las Lagunillas s/n, 23071 Jaen, Spain; irene112189@gmail.com (I.C.-P.); eobrero@ujaen.es (E.O.-G.); 2 Department of Psychology, University of Almeria, Ctra. Sacramento s/n, 04120 Almeria, Spain; 3 Center for Neuropsychological Assessment and Rehabilitation (CERNEP), Ctra. Sacramento s/n, 04120 Almeria, Spain

**Keywords:** stroke, immersive virtual reality, conventional physiotherapy, balance, gait, falls risk

## Abstract

Stroke is a neurologic disorder considered the first cause of disability worldwide due to motor, cognitive, and sensorial sequels. Balance dysfunctions in stroke survivors increase the risk of falls and physiotherapeutic rehabilitation is essential to reduce it. Virtual reality (VR) seems to be an alternative to conventional physiotherapy (CT), providing virtual environments and multisensorial inputs to train balance in stroke patients. The aim of this study was to assess if immersive VR treatment is more effective than CT to improve balance after stroke. This study got the approval from the Ethics Committee of the University of Almeria. Three chronic ischemic stroke patients were selected. One patient who received 25 sessions of immersive VR intervention for two months was compared with another patient who received equivalent CT and a third patient with no intervention. Balance, gait, risk of falling, and vestibular and visual implications in the equilibrium were assessed. After the interventions, the two patients receiving any of the treatments showed an improvement in balance compared to the untreated patient. In comparison to CT, our results suggest a higher effect of immersive VR in the improvement of balance and a reduction of falls risk due to the active upright work during the VR intervention.

## 1. Introduction

Stroke is considered the first cause of disability [1] and the third cause of death in westernized countries after cardiovascular diseases and cancer [2]. Stroke is a central nervous system disorder produced by a local interruption of the cerebral blood flow due to the occlusion (ischemic stroke) or rupture (hemorrhagic stroke) of a cerebral blood vessel [3]. As result of brain cortex injury, afferent and efferent neural pathways are affected, and motor, sensitive and cognitive functions become impaired. Motor and cognitive impairments observed in post-stroke patients reduce their functional capacity, their personal autonomy [4], and social abilities, which results in intensive care and rehabilitation needs with the subsequent economic burden to society and families [5].

Postural instability or poor balance is a relevant central vestibular symptom in neurologic disorders, such as stroke [6], in which approximately 83% of stroke survivors show balance impairments [7]. Proprioceptive visual and vestibular inputs to the central nervous system are essential to guarantee the upright position [8]. Thus, errors in the central integration of this postural information can induce gait difficulties with the subsequent increase in risk of falls [9]. In addition, stroke survivors show a number of neurological issues like visual neglect, sensory loss, reduced muscle strength and spasticity, which also increase the risk of fall 1.5–2 times more in post-stroke patients than older adults without brain damage [10]. This results in fractures, tissue injuries, immobility, and psychological fear of falling as additional consequences of falls in stroke patients [11]. Besides, large hospitalization periods due to injury falls are devastating for patient recovery [12].

The use of virtual reality (VR) has been booming during the last decade, becoming a potential tool in the field of stroke rehabilitation [13]. Virtual reality technology works by displaying a set of digital images that allow the user interacts with a virtual environment or situation that is perceived equivalent to the real physical world [14]. VR has been used in neurorehabilitation in order to encourage a higher number of exercise repetitions and their intensity, and enhances motor learning thanks to the quick feedback possibilities and the multisensorial stimulation [15]. This promotes neuronal plasticity, which would the responsible of VR-induced benefits in stroke rehabilitation [16]. Recent studies have shown that immersive VR protocols in a sitting position and Wii exergames (non-immersive VR) improve motor function, balance, and gait in stroke patients in comparison with conventional therapy (CT) [17]. However, other studies report no statistical differences when comparing immersive or non-immersive VR in a sitting position with CT [18,19]. Moreover, several studies suggest that a neurorehabilitation program combining VR and CT produces a greater improvement than each treatment separately [20]. 

Nevertheless, the majority of published works have used non-immersive VR therapies, such as Wii exergames for balance training [21,22]. Recently, improved versions of immersive VR have become available for clinical and research purposes in physical rehabilitation. Thus, immersive VR, thanks to the use of headsets that display 3D digital images that simulate any scenario with high realism, has the capability to make individuals feel as if they’re inside the virtual environment. Moreover, the use of hand-held controllers allows users to interact with virtual elements using their hands as they do real life, allowing exercise repetition, intensity variation, and task-oriented training. Thus, immersive VR postulates as a promising tool for the rehabilitation of motivated stroke patients. The aim of this study is to assess if an experimental protocol based on immersive VR therapy is valid for stroke rehabilitation and produces positive effects in balance and falls risk in comparisons to a CT protocol. For such a reason, two intervention protocols (immersive VR or CT) in comparison with the absence of treatment were tested in three patients diagnosed with ischemic stroke.

## 2. Materials and Methods 

### 2.1. Study Design

A case report design has been used to carry out this research. This study has been approved by the Ethics Committee for Human Research of the University of Almería (reference UALBIO2019/036). Moreover, it has been done following the ethical principles of the Declaration of Helsinki (2013) [23] and the resolution 466/2012 of the National Health Council. All the participants were informed about the research characteristics and gave their informed consent before participation.

### 2.2. Subjects Selection

Participants were recruited from San Juan de la Cruz Hospital (Úbeda, Spain). We used a non-probability convenience sampling to select the potential participants. Researchers contacted by phone with them, with their family or caregivers to schedule a previous interview and give them information about the study. All potential participants were screened to determine their eligibility according to the inclusion and exclusion criteria. This initial screening was carried out by one of the researchers. 

The inclusion criteria were: (1) Patients older than 18 years and able to provide consent; (2) diagnosis of acute stroke confirmed by neurological assessment including neuroimaging (MRI or computed tomography) within the last 12 months; (3) a slight to medium motor and functional upper limb affection according to the Fugl-Meyer upper extremity scale (score 25–45); (4) absence of cognitive impairments; (5) capability to maintain balance in standing position for ten minutes even with difficulties or needing technical aids; (6) be able to follow verbal instructions related to the use of VR devices; and (7) at least three months have elapsed after the stroke. The exclusion criteria were: (1) new stroke episodes during the rehabilitation process; (2) other issues that could result in disabilities or functional limitations; (3) absence of movement in upper limbs; (4), a score above 9 for the upper limb according to VAS for pain assessment; (5) patients with other neurological conditions such as Alzheimer’s, Parkinson and others dementias; and (6) previous history of epilepsy or neurosurgery. 

Following patient enrollment, we performed the baseline assessment. Then two of the patients were randomly allocated to immersive VR therapy or CT therapy. Treatment allocation was done by an external person who was invited to participate using a random sequence generator. For ethical reasons, the third condition, in which the patient would not receive any treatment for the whole period of the study, a third patient who was not receiving any treatment was chosen to check the possible effects of the absence of therapy. This patient participated just during the baseline and post-intervention assessments. Therefore, our study was conducted with three patients (mean age of 49.33 ± 3.29 years old) who had suffered a left ischemic stroke in the last ten months. Table 1 shows the sociodemographic characteristics of participants.

#### 2.2.1. Patient 1

A 45-year old male diagnosed with left ischemic stroke. After six months of conventional rehabilitation, five days per week, this patient was included in our study. In the first assessment the patient showed a moderate spastic hypertonia in the upper right limb with flexor pattern and functional limitation. In the lower right limb, slight knee flexion with moderate spastic hypertonia, external rotation of the hip, and slight equine foot with steppage gait.

#### 2.2.2. Patient 2

A 50 year-old male diagnosed with left ischemic stroke. This patient was included in our study after nine months of evolution. Before the inclusion, this patient was performing a CT protocol four days per week. The patient showed spastic hypertonia in the upper right limb with shoulder pain and slight elbow flexion, wrist supination and slight finger flexion. The use of feet orthosis was necessary to avoid the equine foot deformity as well as a walking stick to help the gait and balance.

#### 2.2.3. Patient 3

A 53 year-old male diagnosed with left ischemic stroke ten months ago. Due to personal and family circumstances this patient had not received any treatment after hospital discharge. At the time of the study, a certain level of improvement was observed as consequence of the progression and natural course of the disease. The subject was well oriented in spatial and temporal domains. At the beginning of the study, this patient showed high motor and sensory dysfunctions affecting the whole right arm, with a high level of spasticity. The right lower limb was seriously affected as well, showing a marked knee flexor retraction and equine foot with orthosis. This patient did not receive any treatment during the current study and was included to check the potential negative consequences of the absence of therapy.

### 2.3. Intervention Protocol

A neurorehabilitation program based on immersive VR was designed and carried out during a period of two months. A total of 25 sessions (3 alternate sessions per week of 45 min each) were performed with the first patient, including a non-treatment first session for getting the patient used to the VR devices. A physiotherapist supervised and guided the patient during the therapy. 

Some devices were used to develop our neurorehabilitation protocol. As immersive VR setup we choose the *HTC Vive* system composed by a VR headset, two hand-held controllers and two base stations for movement tracking. A laptop “Asus ROG GL502VS-GZ, i7, Nvidia GTX 1070” was used to run the virtual reality applications. With regard to the software, four VR games were used, one week each, in the following order. First, Snaefellsjoekull National Park Iceland from The Lab (Valve™) is a VR game that takes the patient to a mountain landscape where he can also interact with at a robot dog. The next game was Solar System included as well in The Lab. In this case the standing patient must touch and move the virtual planets. In the third game, Climbing Wall from Carnival Games ^®^, the patient must climb a rock wall using his hands and overcome some obstacles. Finally, a virtual kitchen scenario named Ikea Kitchen was used. There, the patient had to cook pancakes and train fine motor and coordination skills. This patient was in a standing position for all gaming conditions. 

The research team met before the study began in order to analyze games characteristics and define the intervention protocol.

### 2.4. Conventional Physiotherapy

Patient 2 followed a CT protocol based on passive or active mobilizations, stretching, proprioceptive and strength exercises, transfers and walking training in the parallel bar. This protocol was performed for a period of 8 weeks in 3 alternate sessions per week of one hour each. This work was conducted in an external physiotherapy center by a member of the research team.

### 2.5. Study Variables

The main variables assessed in these patients were balance, gait, risk and fear of falling and the perception of verticality. Data collection and variable assessment in each one of the three patients were made before the treatments began and right after finishing them. Thus, only immediate changes were observed in this study. The outcome assessor was blind to treatment and this was the first and only interaction between this evaluator and the included patients.

Postural balance was assessed with the Berg balance scale (BBS) [24,25] and the Tinetti scale [26]. The BBS is a reliable and valid scale (with inter and intra-rater reliability of 0.97 and 0.98, respectively) [27] that assess static and dynamic balance in stroke patients. It is composed by 14 items, where each item scored on a 4-point ordinal scale from 0 to 4, and the maximum possible score is 56 points. High scores on this scale indicate a better level of balance [28] and low values indicate a high risk of falls [24]. In addition, the Tinetti Scale was used as a reliable and valid tool to measure balance and gait abilities in patients with stroke with an inter-observer reliability of 0.95 [26]. This scale is composed by two subscales (balance and gait) and the maximum score is 28 points (16 for balance and 12 points for gait) indicates the best possible balance and gait score in stroke patients [26]. 

Objective data regarding the involvement of the visual and vestibular systems in balance and perception of verticality were collected using the Subjective Visual Vertical Test (SVV) [29,30] and the Romberg test [31]. In the SVV test the subjects are asked to adjust vertically a luminous line on a dark background without any cues [32]. This test assesses the contribution of the vestibular system (otolith function) to the sense of verticality. Deviations of verticality above +/−2.5 degrees are considered pathological [30]. The SVV instrument has a high index of usability for patients and an intraclass correlation coefficient of 0.85 (CI = 0.75–0.92) [29]. On the other hand, the Romberg index was used to assess the involvement of different inputs in the postural control of patients with stroke [33]. A stabilometric platform was used to carry out this test, which is accepted as a reliable tool for assessing postural control in neurologic disorders [33]. The mean value is 249 (CI 95% 112–677), lower values indicate a visual dependence and higher values a vestibular dependence.

The timed get up and go test (TGUGT) is a valid instrument [25] with an excellent test-retest reliability (ICC > 0.95) for patients with stroke, and was used to assess gait ability [34]. The TGUGT assesses the time spent by the patient to get up from a chair, then walk three meters, wall back and sit down again [35]. This test was repeated three times to determine a mean test time. Scores lower than 20 s are considered normal but scores above 20 s are related to an increase of falls risk [35]. 

Finally, the falls risk was assessed with the activities-specific balance confidence scale (ABC) [36] and the falls efficacy scale (FES-I) [37]. The ABC scale has a high internal consistency (Cronbach’s α = 0.916), a test-retest reliability of 0.86 (CI 95% = 0.74, 0.93) [36] and has been used in stroke patients [38]. It is a 16-item questionnaire that assesses the level of confidence to maintain the balance. Lower scores are related with a low confidence to maintain balance and an increase in the falls risk [39]. FES-I is a short and standardized tool that measures the fear of falling. It has a high internal consistency, with a Cronbach’s α=0.96 and an intraclass correlation of 0.96 [37]. It is composed of 16 items related to usual daily living activities and its score ranges from 16 to 64 points, with higher scores indicating a greater fear of falling or a lower fall-related self-efficacy [40]. 

## 3. Results

Following patient recruitment, one week before the beginning of the intervention, a blinded researcher collected baseline data (T0) from the three patients. The final evaluation (T1) was conducted just at the end of the treatments by the same researcher. Table 2 summarizes the outcomes for each variable, both before and after the intervention.

### 3.1. Postural Balance

The initial assessment showed that functional postural balance was strongly altered in all three patients. In the post-treatment assessment a higher improvement was observed for patient 1 treated with immersive VR (Berg scale: 22% and Tinetti scale: 72%) and patient 2 who received CT (Berg Scale: 20% and Tinetti Scale: 29%) in comparison with the condition of no treatment which did not show any improvement (12% of deterioration according to the Berg Scale and no change in the Tinetti scale). Regarding the perception of visual verticality, measured with the SVV test (SVV is considered clinically altered for values of ±2.5 degrees), a smaller error was observed in the third patient (with longer evolution), suggesting a vestibular adaptation phenomenon. In both treated patients an improvement of SVV scores was observed but still within pathological levels. The Romberg test showed a vestibular dependence for the first patient and a visual dependence for the second patient. Both interventions improved the Romberg ratio (7% after VR intervention and 17% after CT).

### 3.2. Gait

Following the treatments, improvements of 21% and 7% were found in get up and go test in those patients treated with VR and CT, respectively. However, both patients required more than 20 s in both pre- and post-intervention assessments. Therefore, the risk of falls remained high for all patients, but lower for the patient exposed to immersive VR.

### 3.3. Risk of falls

The intervention based on immersive VR improved balance related to risk of falls by 33% and reduced the fear of falling by 15%. The patient treated with CT also improved his balance (15%) and reduced the fear of falling. Nevertheless, the patient exposed to immersive VR showed a larger improvement for risk of falls balance as well as a higher reduction of fear of falling. In contrast, the patient who did not receive any treatment did not show changes in balance or fear of falling. 

## 4. Discussion

This work is the first experimental case report study that uses an immersive VR protocol composed by a series of immersive VR games as a therapeutic tool for balance disorders in chronic stroke patients. In comparison with other modalities of VR (semi-immersive and non-immersive) such as robotics or Wii games where the patients are trained in a sitting position, our study is based on an immersive VR training protocol which was conducted with the patient in a standing position, increasing the global neuromuscular response. In this sense, previous published studies have used the Wii exergaming to train balance and motor function in stroke patients [21]. Although these studies have shown positive and interesting results, our data suggest that training balance and the global motor function in a standing position is also effective to improve balance after stroke. Besides, this study highlights the importance of training balance in addition to sensorimotor functions to reduce the risk of falling in stroke patients. Moreover, our results show the contribution of visual and vestibular inputs for balance in stroke patients. The aim of our study was to assess if an experimental protocol of isolated immersive VR is more effective than CT to improve postural control and to reduce the risk of falling in chronic stroke patients. We hypothesized that active and continued VR therapy can induce improvements in balance capacity of stroke survivors due to their high engagement and the immersion during the therapy process. 

Stroke is one of the most common causes of impairment for motor function and gait in adults [41], which also depends on the location and severity of brain damage. The main motor issues in stroke survivors are movement limitations, changes in muscle tone, spasticity and impaired motor control [42]. In addition to motor dysfunctions, sensory functions such as visual and vestibular inputs are altered in stroke [43]. As a result of this, stroke survivors experience imbalance due to muscle instability or altered proprioception, decreased postural control, poor voluntary control, misperception of verticality sense [44], and body misalignment [45]. Different studies, including ours, confirm that balance is altered in stroke patients, resulting in a postural control deficit [46,47]. Related to this global balance reduction, falls are the leading cause for the loss of independence observed across stroke patients [48]. Thus, the main factor associated to falls is the loss of balance or balance impairments due to vestibular symptoms such as the misperception of visual verticality [49]. Our results confirm that chronic stroke patients suffer postural instability and present a high risk of falls. Thus, our patients have experienced more than one fall and injury lesions since the stroke episode. On the other side, until the beginning of the study, conventional physiotherapy, as neurorehabilitation strategy, was administered to two of our patients, with the exception of patient 3. 

The aim of neurorehabilitation is to find interventions that accelerate neurological recovery by inducing changes in the post-stroke brain aimed to restore motor and cognitive functions and improve balance related skills. Brain plasticity is a complex mechanism by which the brain remains plastic after stroke [50]. In order to maximize brain plasticity, a great variety of therapeutic approaches have been proposed as neurorehabilitation tools in stroke patients. Thus, classic therapeutic techniques, such as repetitive training exercises, motor therapy, Bobath therapy, or brain imaginary, have been used. However, during the last decade the development of new technologies has promoted the emergence of new therapeutic tools that could help to boost brain plasticity processes and enhance functional recovery [51]. VR has already shown a positive impact in motor recovery after stroke. It has been postulated that VR contributes to neurorehabilitation thanks to its capability to create subjective experiences of immersion [19] and presence during the rehabilitation process [52]. Patients can interact with the virtual environments through a variety of tools like screens, platforms or joysticks (non-immersive VR) or head mounted displays, cameras, sensors and haptic controllers (immersive VR). In addition, therapies based on immersive VR provide a higher level of motivation and adherence to the treatment, although depending on the modality of VR patients become more or less involved in the therapy. 

The vast majority of review [19] and original studies [53] have assessed the effect of non-immersive VR or exergaming in stroke patients. The main variables analyzed have been upper and lower limb functionality, resulting in controversial clinical results. Some authors have reported that non-immersive VR therapy improved upper and lower limb functions, speed gait and global motor functions but did not seem to be superior to CT [54]. However, a combination of VR and CT would result in more significant improvements [19]. We have employed immersive VR as election therapy to test if this kind of VR, more engaging than traditional non-immersive VR tools (Kinect, Wii or exergaming), can improve balance function in stroke patients, comparing the outcome with CT. Immersive VR provides a higher level or realism and interaction during the neurorehabilitation procedure than non-immersive VR. The realistic 360º environment produced by this tool makes possible the patient receives and integrates visual, vestibular and somatosensory information to maintain the balance control. Currently, any study has analyzed balance and risk of falls after a treatment based on immersive VR. Thus, all patients showed balance impairments and high risk of fall according to the Berg Scale, the ABC Scale, and the FES-I Scale scores, but after the intervention procedure, the patient who received immersive VR therapy showed a greater improvement of balance compared with the patient receiving CT. This result was accompanied by a larger reduction of the risk of falling in the VR patient in comparison with the CT patient. The VR patient also experienced a higher walking speed than the others two. However, the risk of falls remained high in all patients. 

VR can be implemented as a new concept of visual and vestibular rehabilitation in stroke patients. We must bear in mind that visual and vestibular information are essential to maintain a correct upright posture [55], so we assessed the vestibular contribution to the sense of verticality [56]. Visual verticality emerges as a concept to understand how the brain creates the perception of body verticality and balance [57]. The brain generates an estimated body schema and peripersonal space. When areas of the visual and vestibular cortices are damaged after the stroke the brain fails to calculate the correct body position, producing a loss of balance [30]. In line with the literature [44], our patients showed a misperception of visual verticality, assessed with SVV test, which revealed a possible vestibular affectation. This vestibular deficit along with visual disturbances and muscle tone disorders are responsible of the loss of balance and the high risk of falls [49]. Vestibular dysfunctions, and visual and verticality impairments observed in stroke patients may be produced by an alteration of the vestibulo-ocular reflex [58] and vestibulo-spinal pathways [59]. Different authors have suggested that VR can improve the adaptation of vestibulo-ocular and vestibulo-spinal [60] reflexes. In addition, several studies have suggested that balance training in a standing position can be modulated by the combined activity of vestibulo-spinal and vestibulo-ocular reflexes, due to the increased demand for maintaining the postural control [61]. According to this, training stroke patients balance immersed in a multisensory VR environment in an upright position would positively involve the vestibular contribution for maintaining the upright posture [62]. Immersive VR may induce a recovery or adaptation of vestibular and ocular functions responsible of balance [63] and reduce the risk of falls [64]. Therefore, this can be a new approach for treating balance impairments related to vestibular dysfunctions in stroke patients.

As a final consideration, we have to mention that our research had been conceived as a pilot study and therefore it has some drawbacks. The most relevant limitation is that our work is a case report study with only one representative case for each treatment, so the generalization of the results must be cautious. Therefore, there is a potential selection bias in our study that affects the external validity of the results. In order to assess immersive VR effectiveness, it will be essential to increase the sample to avoid any selection bias and to get a representative sample size with the aim of increasing the validity and robustness of our results. Another limitation is that only ischemic stroke patients have been tested and the absence of statistical analyses due to the small number of participants. Therefore, the use of a probabilistic sampling method would be required. Another limitation is that post-treatment assessments were carried out immediately after the interventions, so a therapeutic prognosis cannot be established. Although patient allocation was randomized for the two possible treatments, we consider that the small sample size increases the potential effect of confusing variables. Moreover, we must acknowledge a selection bias issue regarding the allocation of the untreated patient. Therefore, outcome comparisons between the untreated participant and patients receiving treatment should be taken with extreme caution. On the other hand, periodical assessments beyond the immediate end of the treatment must be included to check medium- and long-term effects. This will permit to know whether these results are temporary or permanent. However, we believe that the present study is useful and can help to establish new protocols of immersive VR therapy where stroke patients can train balance and gait in a standing position.

## 5. Conclusions

Compared to CT, our results suggest a higher effect of immersive VR improving balance and reducing the risk of falls, as well as the perception of visual verticality in stroke patients. CT also produced an improvement of balance and reduced the risk of falls, whereas the absence of treatment resulted in a decrease of balance and a subsequent loss of functional capacity. We consider that immersive VR can be postulated as a valuable tool for treating balance impairments in chronic ischemic stroke. Nevertheless, this tool is also compatible with the use of CT so both strategies can be used in combination. However, due to low sample size, it is convenient to consider important selection biases that may affect the external validity of the results and its generalization. Future studies must reduce and control these biases in order to enhance the quality and robustness of the findings. Our study can be a worthy starting point for balance rehabilitation in stroke patients by using a standing position.

## Figures and Tables

**Table 1:** Main characteristics of patients included in the study.

CHARACTERISTICS		Participant 1	Participant 2	Participant 3
**Socio-demographic**	**Age**	45	50	53
	**Gender**	Male	Male	Male
	**Weigh (kg)**	73	84	65
	**Height (cm)**	175	179	177
	**Body Max Index**	23.8	26.2	20.8
	**Marital status**	Married	Married	Divorced
	**Education level**	University	Primary	Primary
**Prior comorbidity**	**Hypertension**	Yes	Yes	Yes
	**Hypercholesterolemia**	No	No	Yes
	**Diabetes mellitus**	No	Yes	No
	**Vertigo**	Yes	No	No
	**Falls**	Yes	Yes	Yes
	Number of falls *	2	3	1
	**Injuring falls**	Yes	Yes	Yes
	**Type of injury**	Hematoma	Wounds	Bone fracture
**Diagnosis**	**Stroke type**	Ischemic	Ischemic	Ischemic
	**Stroke localization**	Left	Left	Left
	**Hemiplegia**	Yes	Yes	Yes
	**Side**	Right	Right	Right
	**Evolution**	6 months	9 months	10 months
**Assigned intervention**		Immersive VR	CT	NI

VR = Virtual Reality; CT = Conventional Therapy; NI = Not Intervention. * Number all falls after stroke.

**Table 2:** Main findings in patients.

VARIABLES		OUTCOMES											
INTEREST VARIABLE	RATING SCALE	P1 (VR)				P2 (CT)				P3 (NT)			
		T0	T1	% var	Clin Imp.	T0	T1	% var	Clin Imp.	T0	T1	% var	Clin Imp.
**BALANCE**	**BBS**	28	34	22	Yes	15	18	20	Yes	8	7	12	No
	**Tinetti**	11	19	72	Yes	7	9	29	Yes	6	6	0	No
**VESTIBULAR**	**SVV**	5.75 ± 1.25	4 ± 1.2	30	Yes	5.2 ± 2.7	4.8 ± 1.8	7	Yes	3.7 ± 2	3.4 ± 1.67	8	Yes
	**Romberg**	427	398	7	Yes	192	226	17	Yes	NP	NP	NP	NP
**FALLS**	**ABC**	24	32	33	Yes	13	15	2	Yes	4	4	0	No
	**FES-I**	47	40	15	Yes	59	54	8	Yes	61	60	2	Yes
**GAIT**	**TGUGT**	29	23	21	Yes	33	31	7	Yes	NP	NP	NP	NP

P = Participant; VR = Virtual Reality; CT = Conventional Therapy; NT = Participant did not have been assisted; T0 = Baseline evaluation; T1 = Final evaluation; Clin Imp = Clinical Improvement; % var = percent change between T0 and T1; BBS = Berg Balance Scale; SVV = Subjective Visual Vertical; ABC = Activities Specific Balance Confidence; FES-I = Falls Efficacy Scale; TGUTP = Timed Get Up and Go Test; NP = Not possible to assess.
